# Prevalence and distribution of *Varroa destructor* and *Nosema* spp. in symptomatic honey bee colonies across the USA from 2015 to 2022

**DOI:** 10.1038/s41598-024-51514-9

**Published:** 2024-01-19

**Authors:** Samuel Abban, Bart Smith, Miguel Corona, Steven C. Cook, Jay D. Evans, Yanping Chen, Mohamed Alburaki

**Affiliations:** USDA-ARS Bee Research Laboratory, Beltsville, MD 20705 USA

**Keywords:** Parasitology, Pathogens

## Abstract

USDA-ARS Bee Research Laboratory received symptomatic honey bee (*Apis mellifera* L.) samples across the United States for disease diagnosis. Here, we present a retrospective study and cartography of ectoparasite *Varroa destructor* and intracellular microsporidia parasite *Nosema* spp. These two major parasites were identified in the diseased honey bee samples between 2015 and 2022. *Varroa* infestation level (VIL) was examined by a wash technique (Mites/100 bees) and calculated as a percentage, while *Nosema* infection was quantified by microscopical spore count (Million Spores/Bee). Data were analyzed by month, year, state, and by nine geographical climate regions described in the U.S. Of adult bee samples (*n* = 4039) that were analyzed for *Varroa* mite infestation, the overall VIL in the U.S. ranged between 0.4 and 30.85%, with an overall national VIL and *Varroa* prevalence of 8.21% and 85.14%, respectively. Overall monthly data showed VIL constantly exceeded the critical level of 4% except from June to September and reached a maximum of 15% in January and December. Nationwide, VIL significantly (*p* < 0.001) increased from 2015 to 2018 (1.1–4.7%), plateaued from 2018 to 2021 (4.7–4.5%), followed by a significant decrease in 2022 (3.6%). Significant VIL differences (*p* < 0.001) were recorded among climate regions, with the highest mite infestation levels in the Upper Midwest region (13.9%) and the lowest in the West region (5.1%). Of adult bee samples (*n* = 2,994) that were analyzed *for Nosema* infection, *Nosema* spore count ranged between (1–16.8) million spores per bee among states, with a national average of 6.8 and a prevalence of 99.7%. The lowest and highest *Nosema* loads were respectively recorded in the South region (3.1) and Upper Midwest (10.5), a significant difference (*p* < 0.001). No statistical differences were recorded among the six other climate regions. Overall, VIL and *Nosema* infection correlated significantly (*p* < 0.001) with a regression coefficient of (R^2^ = 0.6). Our data, which originated from ailing bee colonies, showed significantly higher rates of maladies compared to data from healthy colonies obtained by the USDA-APHIS National Honey Bee Survey, demonstrating the role of bee diseases caused by *Varroa* mite and *Nosema* in honey bee population declines.

## Introduction

European honey bees (*Apis mellifera* L.) are important pollinators, playing a crucial role in upholding food production and maintaining the biodiversity of agricultural ecosystems and the environment^1–3^. In addition to providing pollination service to numerous crops and wild plants, honey bees also produce honey and other hive products, such as beeswax, pollen, propolis, and royal jelly^4^. These products have been used for their nutritional, medicinal, and cosmetic properties for centuries^5^. Although various pollinators exist worldwide, honey bees are the most effective and efficient for large-scale crop pollination due to their remarkable social behavior, ability to communicate with their mates regarding forage locations, and ease of management^6,7^. In the U.S. alone, the value of pollination services provided by honey bees is estimated to be 17 billion dollars annually^8^. Some crops, such as almonds, rely entirely on honey bees for pollination.

The alarming decline in honey bee populations and health in recent years has raised serious concerns about the future security of pollination services for food production^9,10^. Of multiple factors include diseases, pesticide exposure, habitat loss, climate change, and nutrition deficiencies^11,12^ that contribute to honey bee colony decline, diseases caused by invasive pests and emerging pathogens such as *Varroa* mite and microsporidia parasite *Nosema ceranae* pose particular threats to honey bee health and negatively affect their performance and survival.

The ectoparasitic mite *Varroa destructor* feeds on the fat body and hemolymph of honey bees^13^ and serves as a vector to transmit several deadly viruses, causing significant damage to honey bee colonies^14,15^. This parasite was first reported in the U.S. in 1987^16,17^ and has forever changed the U.S. beekeepers since its first detection. Varroosis, a disease caused by *Varroa* infestation, is the most destructive disease for honey bees. The symptoms of varroosis and associated diseases include physical deformities, reduced bee lifespan, weakened immune systems, rapid loss of adult bee population, and even collapse of the entire colony^18–21^. So far, the devastating disease has destroyed millions of *A. mellifera* colonies since its establishment in various parts of the world^22–24^ and obliterated feral colonies from many areas. Control of *Varroa* mite infestations can be achieved through chemical and non-chemical treatments as well as Integrated Pest Management (IPM) approaches that combine both non-chemical and chemical methods^25–27^.

Nosemosis is a serious and common disease of adult honey bee workers caused by intracellular microsporidia parasites in the genus *Nosema*, which are specialized fungi. The recent proposal to reclassify *Nosema* under the *Vairimorpha* genus^28^ has encountered substantial opposition and is presently a focal point of vigorous debate within the worldwide scientific community. Consequently, this study has opted to retain the historically recognized and utilized genus “*Nosema*”. For decades, nosemosis of *A. mellifera* was exclusively attributed to a single *Nosema*, *N. apis* species. In 2005, a natural infection of *N. ceranae*, a *Nosema* species first found in the *A. cerana *^29^, was identified as a disease agent of *A. mellifera* colonies^30^. Shortly after that, the infection of *N. ceranae* in *A. mellifera* was reported worldwide and has been associated with colony losses^31,32^. Currently, nosemosis caused by *N. ceranae* in *A. mellifera* is far more prevalent than that caused by *N. apis*, and *N. ceranae* is replacing its congener *N. apis* in most regions of the world. *Nosema* infection starts with the ingestion of spores via contaminated food. The spores then multiply in the midgut, damaging the gut lining and impairing digestion^33^. The clinical symptoms of *Nosema* infection include dysentery, reduced foraging activity, weakened immune system, and reduced bee lifespan. In severe cases, the infection can lead to colony collapse.

Since its inception in 1891, the Bee Disease Diagnostic Service of the USDA-ARS Bee Research Laboratory (BRL) has been providing a free-of-charge disease diagnosis for beekeepers and other federal and state agencies across the U.S. In this report, we retrieved our historical disease diagnostic results obtained between 2015 and 2022 and conducted a full epidemiological assessment of the frequencies of *Varroa* infestation and *Nosema* infection across the states. We find consistent regional ‘hotspots’ for parasites across years, heavy annual variation, and regional concordance between the results from our diagnostic service of disease-symptomatic colonies versus public results from the National Honey Bee Health survey, which does not focus on symptomatic or failing colonies.

## Results

### Sample tally and distribution

**Figure 1:**
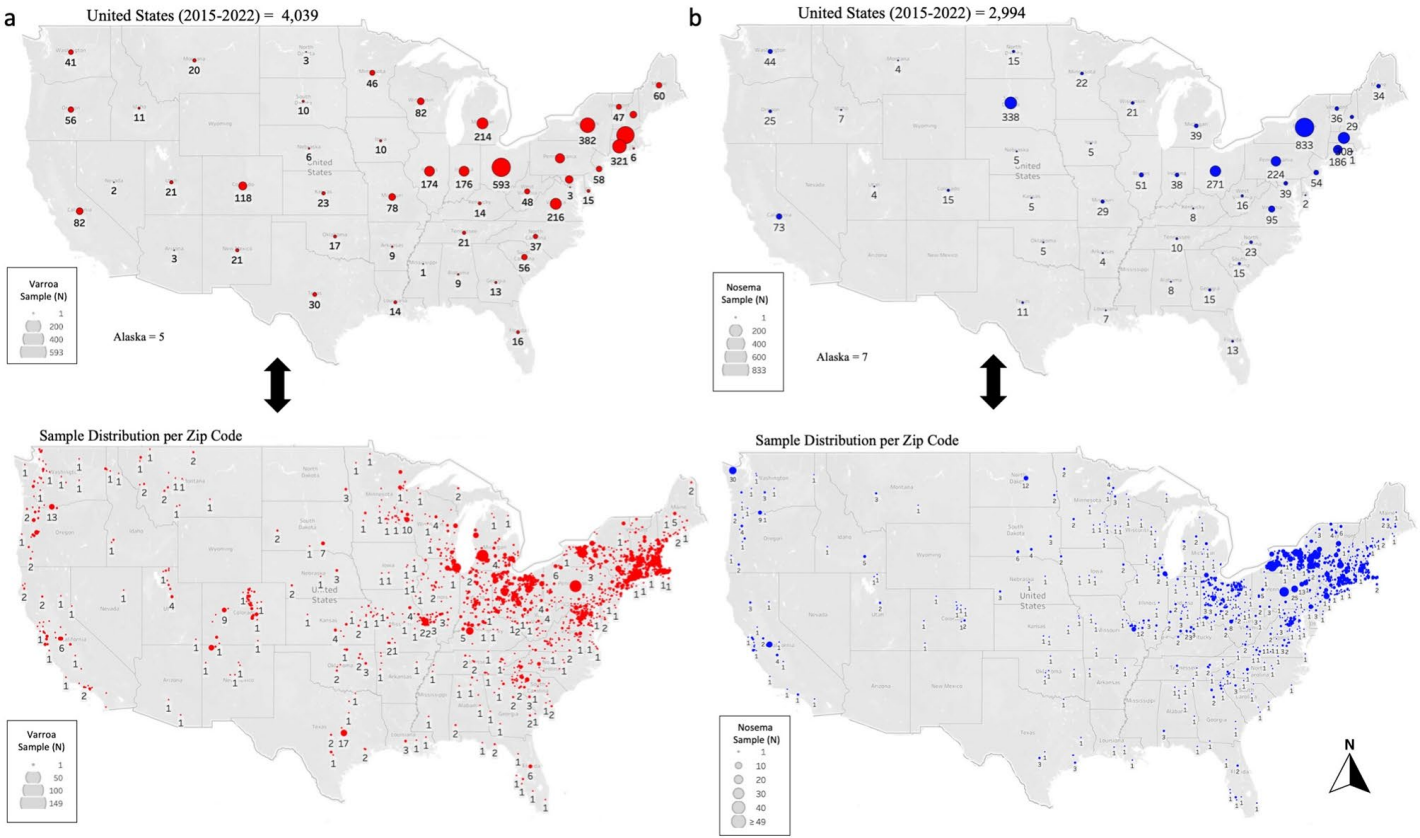
Overall number and distribution of analyzed samples from 2015 to 2022 across the U.S., displayed per state and county Zip code. (**a**) Samples analyzed for *Varroa* mite per 100 bees and (**b**) *Nosema* spore count per bee. Overlapping samples in the Zip code maps were omitted for better visualization of the sample distribution.

During its activity between 2015 and 2022, the Bee Disease Diagnostic Laboratory (BDDL) analyzed a total of 7033 samples, among which 4039 samples were analyzed for *Varroa* mite infestation and 2994 samples for *Nosema* infection via spore count from across the U.S. (Fig. 1 and Table 1). During the same period, we also processed samples sent by State Apiary Inspectors and beekeepers from all fifty states of the U.S. except for Hawaii. The three highest States in terms of sample size contribution were New York (*n* = 1215), Ohio (833), and Massachusetts (829), Table 1. Similarly, states with the lowest number of samples requesting our service were Mississippi (1), Nevada (2), and the District of Columbia (3) (Table 1).

**Table 1:** Overall nationwide and state summary of *Varroa* mite infestation and *Nosema* spore count between 2015 and 2022. N1: Number of analyzed samples per state for *Varroa* mite, N2: Samples analyzed for *Nosema*. T1: Total number of samples analyzed per state. T2: National sums and averages, VP: *Varroa* prevalence, NS: *Nosema* spore count (Million Spores/Bee), and NP: *Nosema* prevalence. States were assigned to climate regions according to the U.S. National Centers for Environmental Information (Karl and Koss 1984).

Code	N1	N2	T1	VIL%	VP%	NS	NP%	Climate Region	Abbrev. CR
AK	5	7	12	1.08	100	9.02	100	–	–
AL	5	8	13	10.69	100	8.84	100	Southeast	SE
AR	9	4	13	7.6	88.9	1.69	100	South	SW
AZ	3	–	3	5.83	100	–	–	Southwest	SW
CA	82	73	155	5.2	65.9	4.76	100	West	W
CO	118	15	133	9.04	83.1	2.77	100	Southwest	SW
CT	321	186	507	10.29	99.4	7.28	99.5	Northeast	NE
DC	3	-	3	5.93	66.7	–	–	Northeast	NE
DE	15	2	17	30.85	100	7.85	100	Northeast	NE
FL	16	13	29	2.81	87.5	3.97	100	Southeast	SE
GA	13	15	28	7	84.6	8.91	100	Southeast	SE
IA	10	5	15	13.21	90.0	6.28	100	Upper Midwest	UMW
ID	11	7	18	6.54	90.9	1.22	100	Northwest	NW
IL	174	51	225	7.86	75.3	6.08	100	Ohio Valley	OV
IN	176	38	214	19.08	93.8	5.87	100	Ohio Valley	OV
KS	23	5	28	6.8	91.3	1.09	100	South	S
KY	14	8	22	8.74	85.7	4.54	100	Ohio Valley	OV
LA	14	7	21	1.4	92.9	3.51	100	South	S
MA	521	308	829	11.2	88.5	7	98.7	Northeast	NE
MD	104	39	143	6.61	85.6	16.84	97.4	Northeast	NE
ME	60	34	94	9.25	88.3	6.96	100	Northeast	NE
MI	214	39	253	17.74	80.8	7.5	100	Upper Midwest	UMW
MN	46	22	68	8.9	89.1	15.77	100	Upper Midwest	UMW
MO	78	29	107	6.4	85.9	10.99	96.6	Ohio Valley	OV
MS	1	–	1	0.4	100	–	–	South	S
MT	20	4	24	10.6	85.0	2.36	100	Northern Rockies and Plains	NRP
NC	37	23	60	9.97	97.3	14.12	100	Southeast	SE
ND	3	15	18	11.23	100	3.3	100	Northern Rockies and Plains	NRP
NE	6	5	11	5.5	100	12.05	100	Northern Rockies and Plains	NRP
NH	78	29	107	14.43	93.6	15.18	100	Northeast	NE
NJ	58	54	112	12.28	93.1	10.45	100	Northeast	NE
NM	21	–	21	3.96	47.6	–	–	Southwest	SW
NV	2	–	2	0.5	50.0	–	–	West	W
NY	382	833	1215	10.27	91.1	4.68	99.0	Northeast	NE
OH	593	271	864	6.76	92.6	3.46	100	Ohio Valley	OV
OK	17	5	22	10.97	82.4	0.99	100	South	S
OR	56	25	81	7.44	80.4	3.46	100	Northwest	NW
PA	152	224	376	12.49	89.5	4.39	100	Northeast	NE
RI	6	1	7	2.73	83.3	11.8	100	Northeast	NE
SC	56	15	71	2.54	66.1	1.28	100	Southeast	SE
SD	10	338	348	7.6	100	3.44	99.7	Northern Rockies and Plains	NRP
TN	21	10	31	10.4	90.5	4.15	100	Ohio Valley	OV
TX	30	11	41	2.85	100	5.17	100	South	S
UT	21	4	25	4.51	61.9	8.78	100	Southwest	SW
VA	216	95	311	7.15	82.9	11.92	100	Southeast	SE
VT	47	36	83	3.59	42.6	3.43	97.2	Northeast	NE
WA	41	44	85	3.85	65.9	6.2	100	Northwest	NW
WI	82	21	103	6.9	70.7	11.51	100	Upper Midwest	UMW
WV	48	16	64	13.32	91.7	8.22	100	Ohio Valley	OV
**T2/AVG**	4039	2994	7033	8.21	85.14	6.80	99.73	**–**	**–**

### *Varroa* infestation level

**Figure 2:**
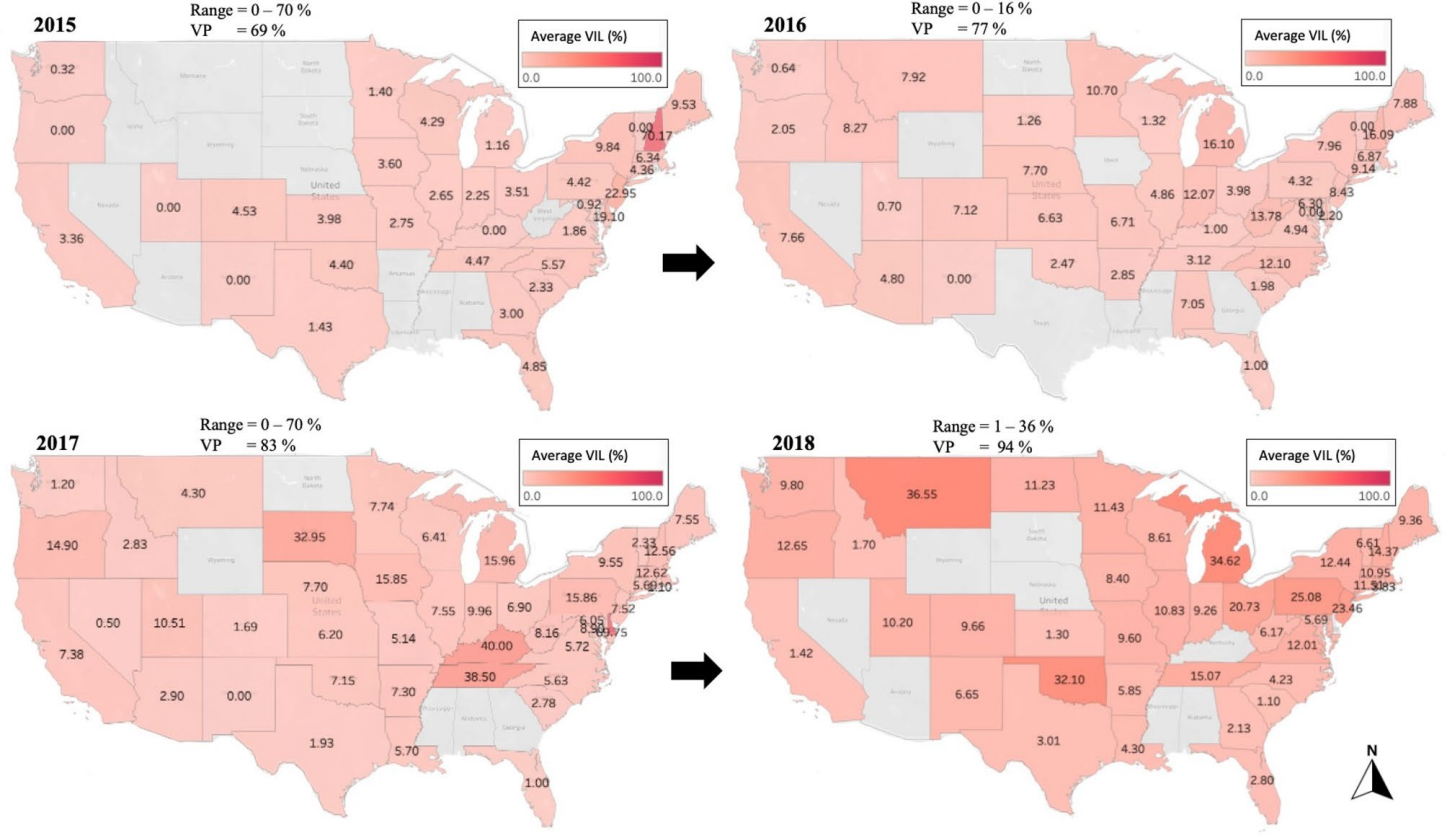
Percentage of average *Varroa* Infestation Level (VIL), displayed per state and year (2015–2018). States with gray font did not send samples for analysis. Mites were counted from adult honey bees using a wash technique described in the materials and methods. VIL range and *Varroa* prevalence (VP) per year are summarized on top of each map.

**Figure 3:**
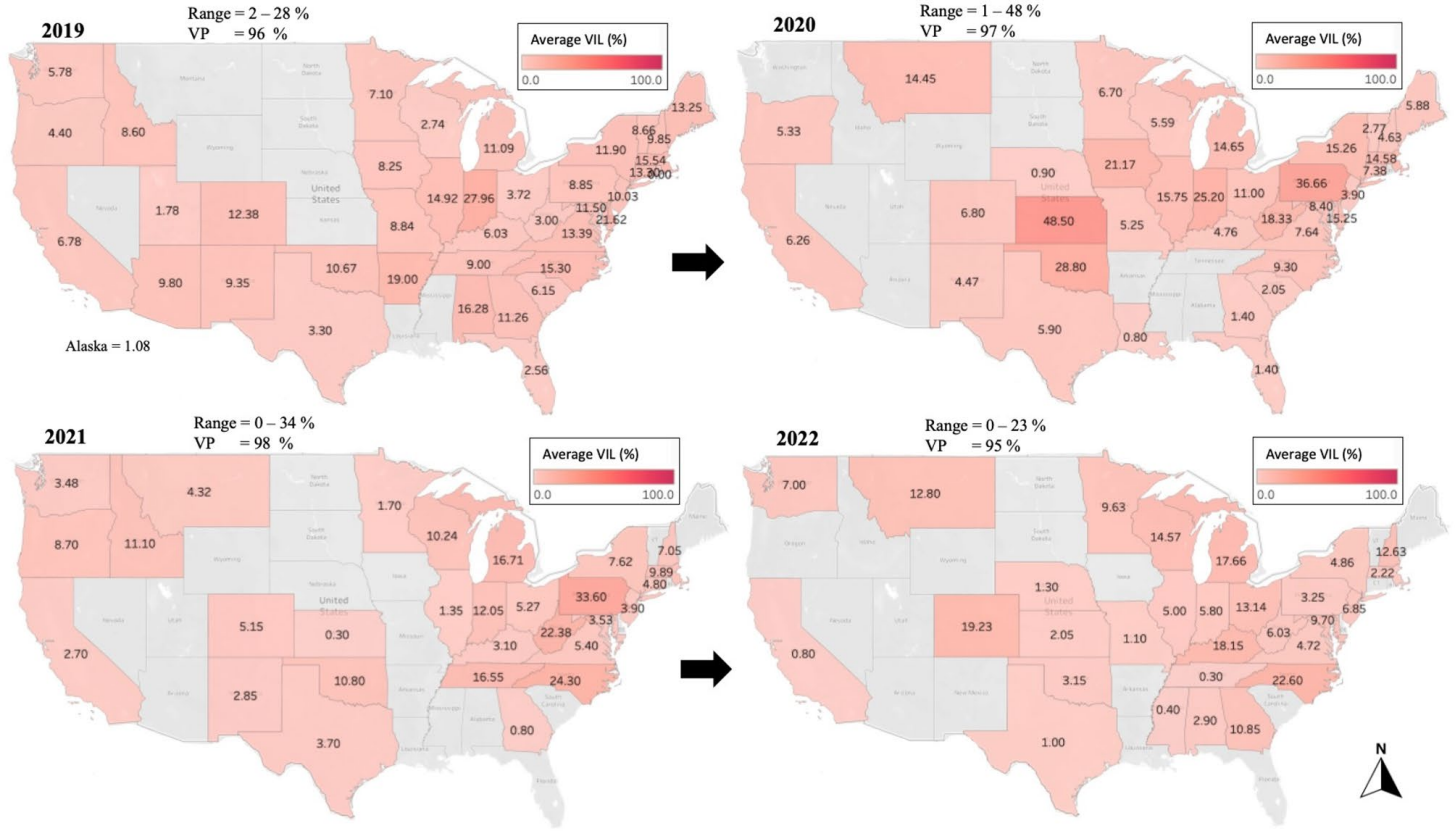
Percentage of average *Varroa* Infestation Level (VIL), displayed per state and year (2019–2022). States with gray font did not send samples for analysis. Mites were counted from adult honey bees using a wash technique as described in the materials and methods. VIL range and *Varroa* prevalence (VP) per year are summarized on top of each map.

**Figure 4:**
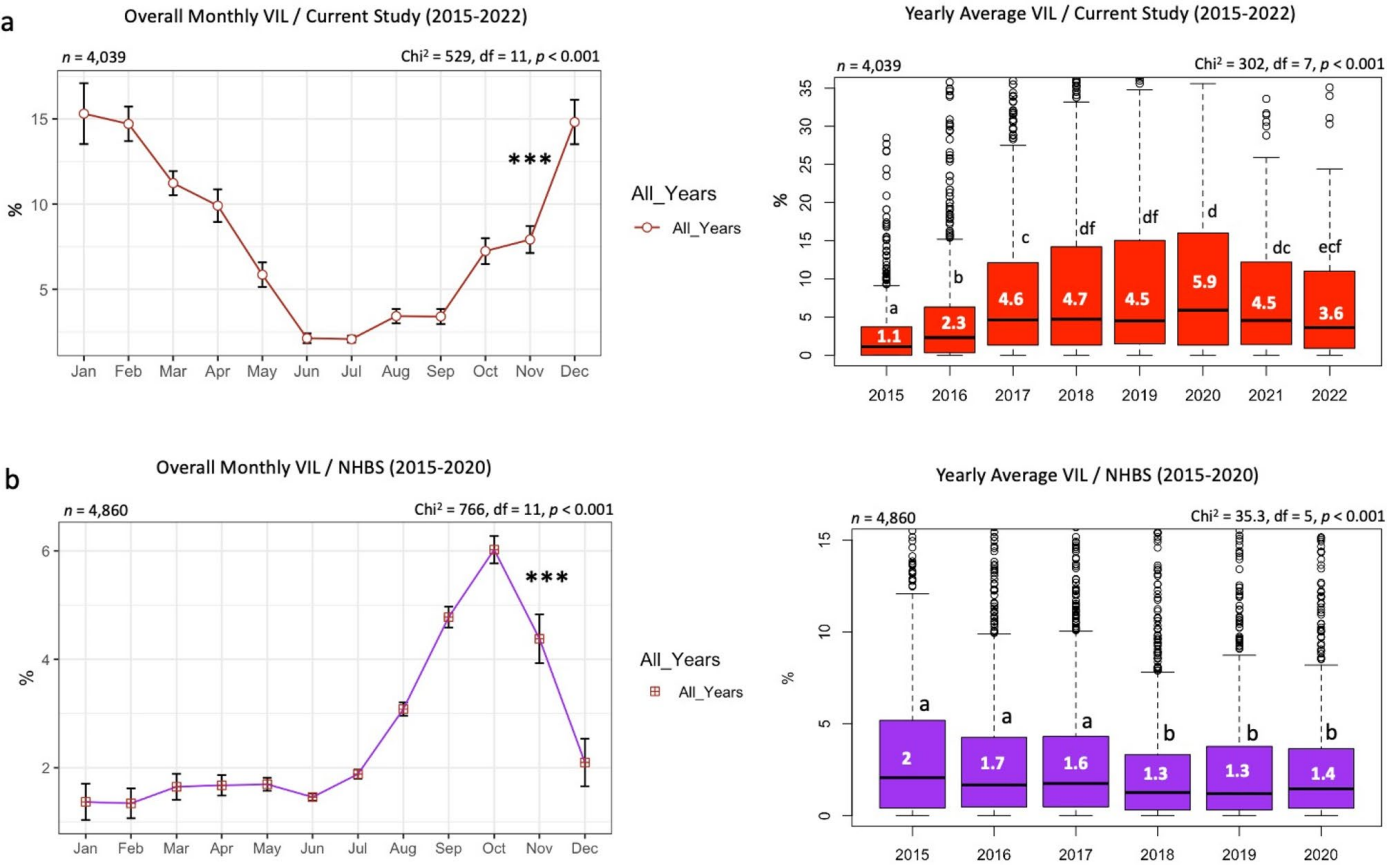
Nationwide averages of *Varroa* Infestation Level (VIL) displayed by month and year for data of the current study (**a**) and the USDA-APHIS National Honey Bee Survey NHBS (**b**). (n) is the number of samples in each graph and statistical analysis. Error bars in the line graphs are the Standard Error (SE). The Kruskal–Wallis test was conducted at three levels of significance: *p* < 0.05*, *p* < 0.01**, *p* < 0.001***. The box plot's median is displayed in white font, and box plots with different alphabetic letters are statistically significant.

Based on the data collected from our analyzed samples, *Varroa* mite infestation level (VIL) varied significantly over the years (Figs. 2, 3). The highest VIL in 2015: NH (70.17%) and NJ (22.95%); in 2016: NH (16.09%) and WV (13.78%); in 2017: KY (40%) and TN (38.5%); in 2018: MT (36.55%) and MI (34.62%) (Fig. 2). In 2019, the highest VIL was recorded in IN (27.96%); in 2020: KS (48.5%); in 2021: PA (33.6%) and in 2022: NC (22.6%) (Fig. 3). Over all years, states that had a lower VIL than (3%) were MS, NV, AK, LA, SC, RI, FL, and TX (Table 1). States with VIL exceeding an overall average of (13%) were DE, IN, MI, NH, WV, and IA (Table 1). The overall monthly mite loads were significantly (*p* < 0.001) different, with a VIL < 5% between June to September (Fig. 4). The highest mite loads were identified in samples sent and analyzed in Winter (Nov to Jan), early Spring (Feb to Apr) and Autumn (Oct-Nov) (Fig. 4a). Overall average of mite infestation showed significant differences (*p* < 0.001) across years (Fig. 4a). The lowest overall VIL was recorded in 2015 with a median VIL of 1.1%, followed by three consecutive significant (*p* < 0.001) increases in mite loads in 2016 (2.3%), 2017 (4.6%), and 2018 (4.7%) (Fig. 4b). No significant differences were recorded in mite load between 2018 and 2021, showing a steady rate with a VIL median range of (4.5–5.9%) (Fig. 4a). In 2022, mite load dropped significantly to 3.6% nationwide (Fig. 4a). The national U.S. VIL and prevalence across states were respectively 8.21% and 85.14% (Table 1). The lowest *Varroa* prevalence (VP) was recorded in VT (42.6%), NM (47.6%), and NV (50%) (Table 1). Similar to our data, the NHBS VIL data showed significant (*p* < 0.001) variations across months, with the highest VIL recorded in October (6%) (Fig. 4b). The yearly VIL showed a steady decrease from 2015 (2%) to 2020 (1.4%) (Fig. 4b).

### VIL and climate regions

**Figure 5:**
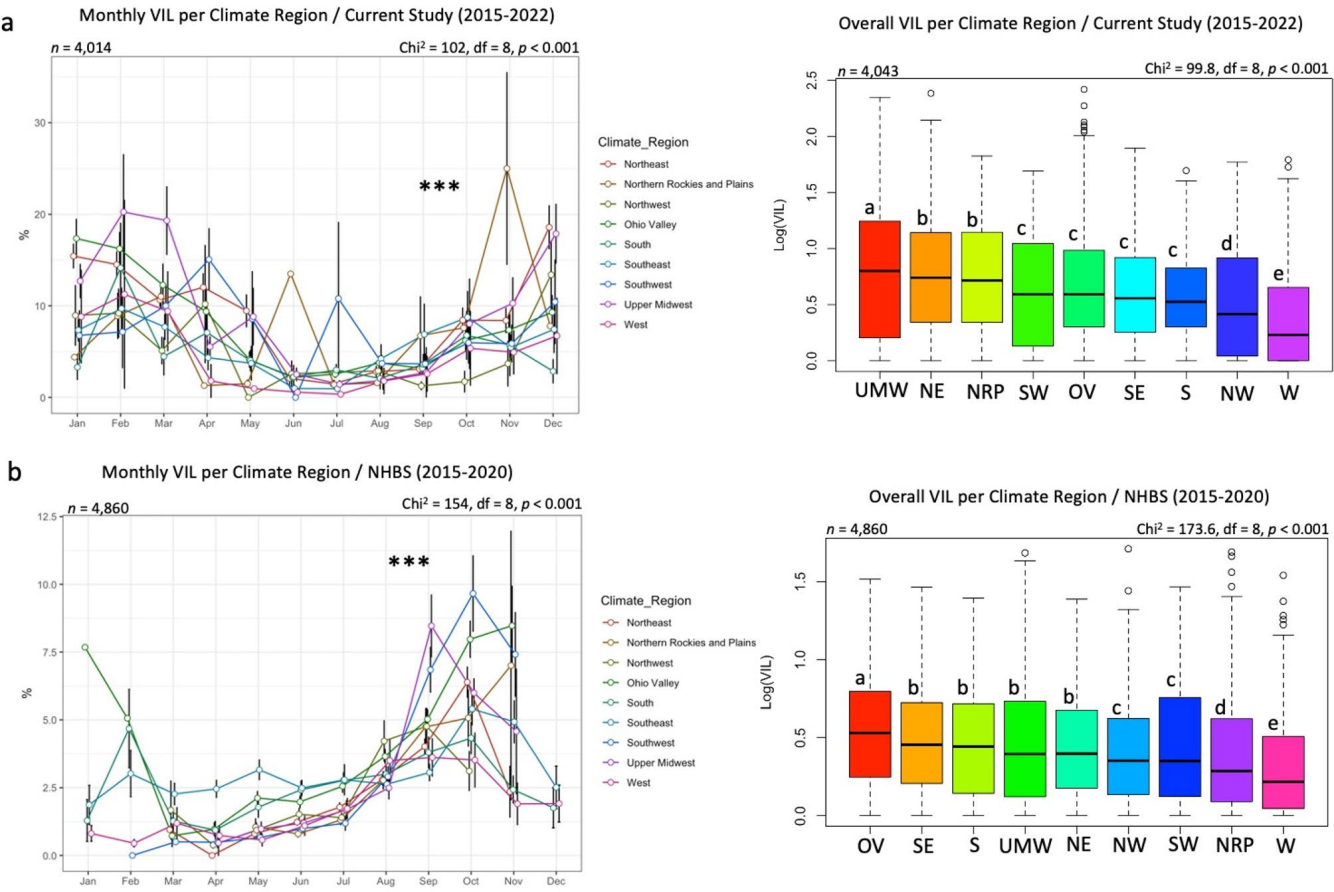
Nationwide *Varroa* Infestation Level (VIL) displayed by climate region per month and overall average on data of the current study (**a**) and the USDA-APHIS National Honey Bee Survey NHBS (**b**). (n) is the number of samples in each graph and statistical analysis. Error bars in the line graphs are the Standard Error (SE). The Kruskal–Wallis test was conducted at three levels of significance: *p* < 0.05*, *p* < 0.01**, *p* < 0.001***. Box plots with different alphabetic letters are statistically significant. Climate region abbreviations are given in Table 1.

Our results evidenced statistically significant (*p* < 0.001) variations in *Varroa* infestations among climate regions, whether per month or as overall averages (Fig. 5). In the monthly load analysis, the lowest elbow of the VIL curves matches the period between June to August. The highest overall VIL was recorded in the Upper Midwest region (IA, MI, MN, WI), while the lowest VIL was documented in the West region (CA and NV) (Fig. 5a and Table 1). VIL of other climate regions ranged between these two ends, with significant inter-region differences detailed in Fig. 5a. Concerning the NHBS data, monthly lower VILs per region are recorded between March to July, with significant increases in Autumn (Sep-Nov) (Fig. 5b). The highest overall VIL in the NHBS data was recorded in the Ohio Valley region, while the lowest VIL was in the West region, similar to what was obtained in our data (Fig. 5b).

**Figure 6:**
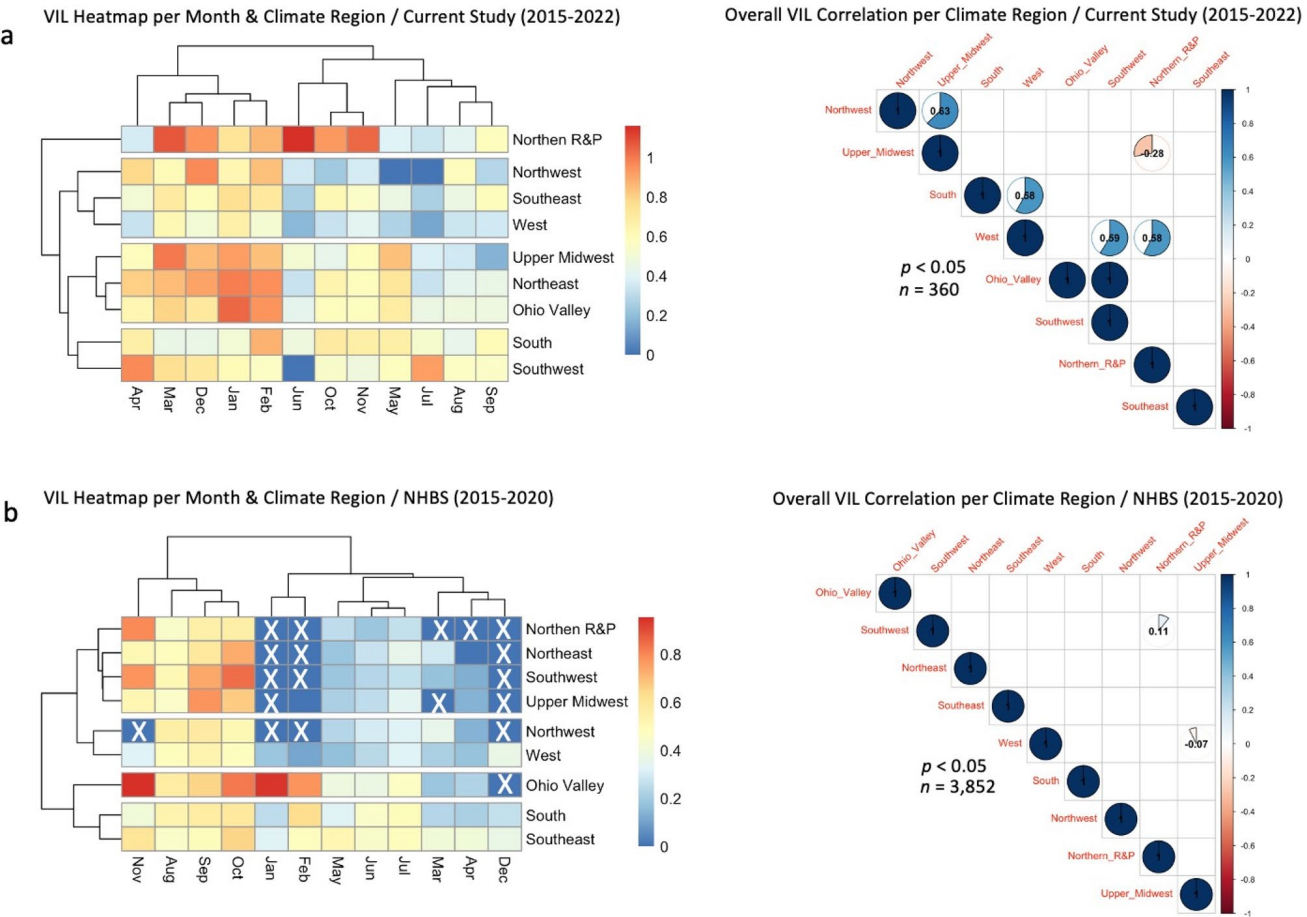
Heatmap and correlation of *Varroa* Infestation level (VIL) per month and climate region. VIL heatmap per month and climate region and correlation of VIL among climate regions on current study data (**a**) and the NHBS data (**b**). White crosses are unavailable values from NHBS data. Correlation analyses were conducted at a cutoff of *p* < 0.05, R-values are given within pairwise correlation circles, only significant correlations are displayed, and blank squares were not statistically significant. The assignment of states to climate regions is detailed in Table 1.

Based on the VIL similarity in our data, the heatmap dendrogram distinguished four groups of regions: 1-(NRP), 2- (NWM, SW, W), 3- (UMW, NE, OV), and 4- (S, SW) (Fig. 6a). Few VIL correlations between climate regions were identified, the strongest being between Northwest and Upper Midwest (*p* < 0.05, r = 0.63) (Fig. 6a). Similarly, the NHBS data showed very few VIL correlations among climate regions with likewise VIL tendency across months between South and Southeast regions (Fig. 6b). Missing winter data from the NHBS heatmap restricts further interpretations.

### *Nosema* infection

**Figure 7:**
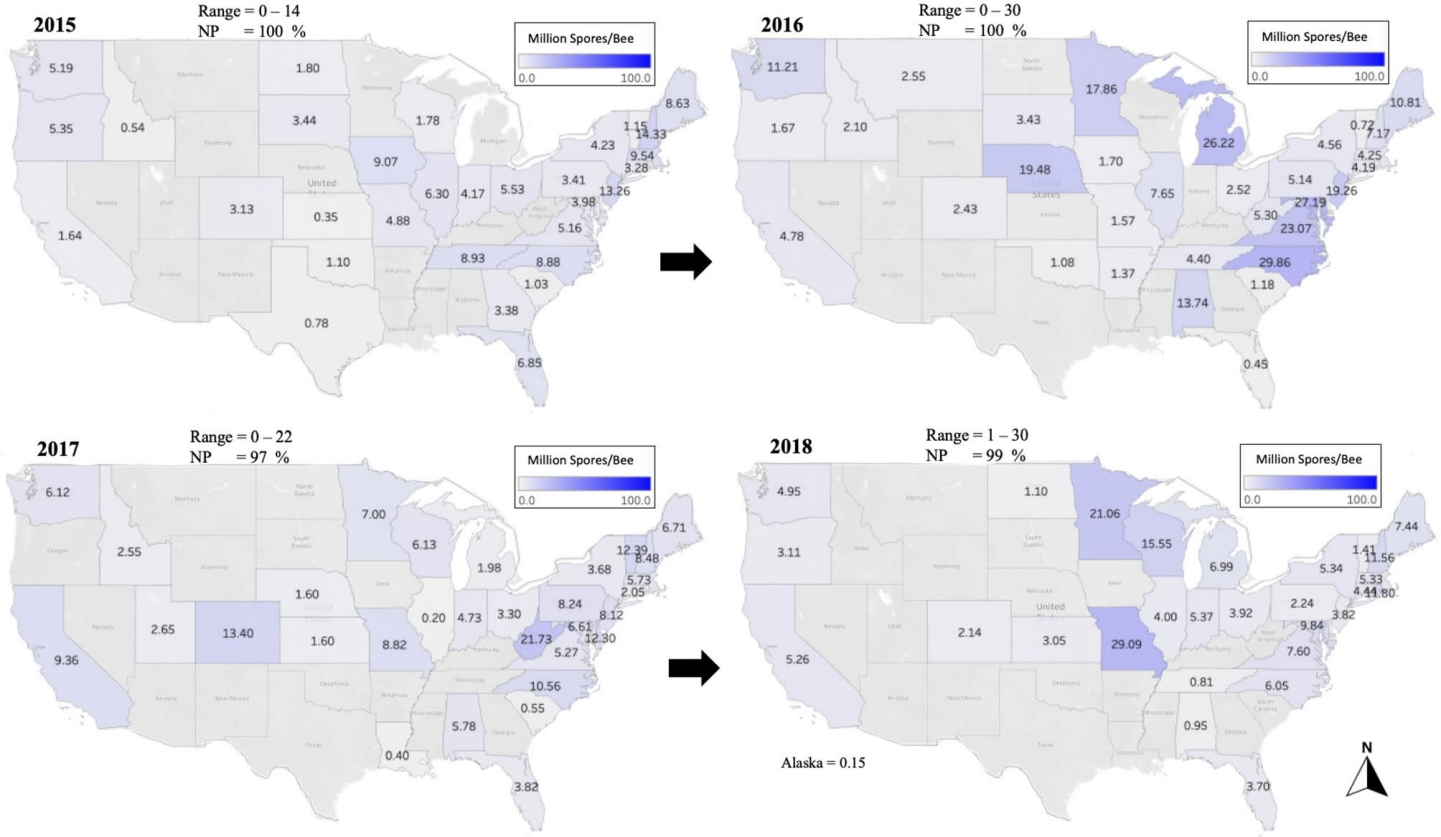
Overall averages of *Nosema* spores identified per bee (Million Spores/Bee), displayed per state and year (2015–2018). States with gray font did not send samples for analysis. *Nosema* spore counts were conducted on adult honey bees as described in the materials and methods. The national range per year is summarized on top of each map. National *Nosema* range and prevalence (NP) per year are summarized on top of each map.

**Figure 8:**
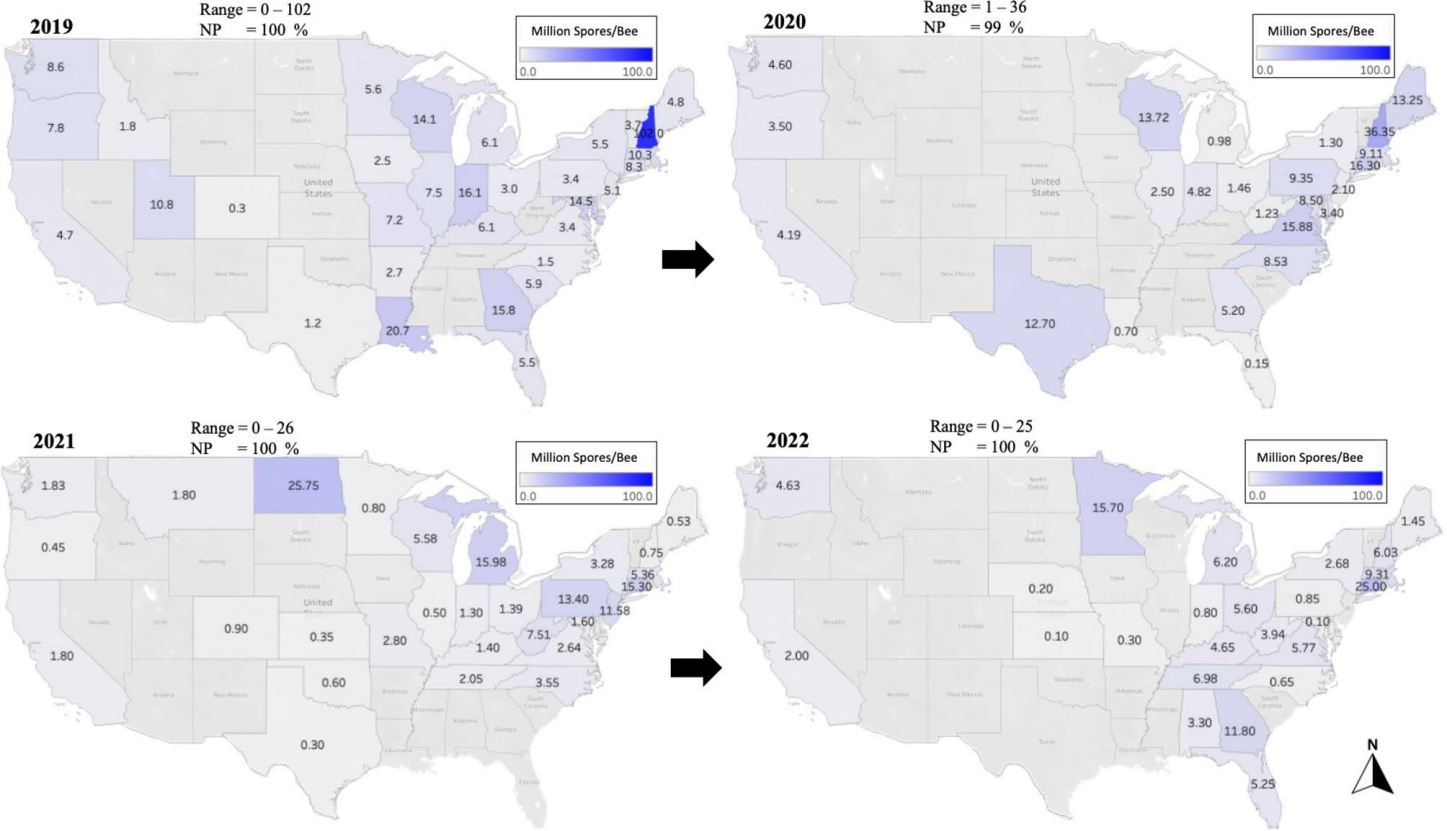
Overall averages of *Nosema* spores identified per bee (Million Spores/Bee), displayed per state and year (2019–2022). States with gray font did not send samples for analysis. *Nosema* spore counts were conducted on adult honey bees as described in the materials and methods. National *Nosema* range and prevalence (NP) per year are summarized on top of each map.

**Figure 9:**
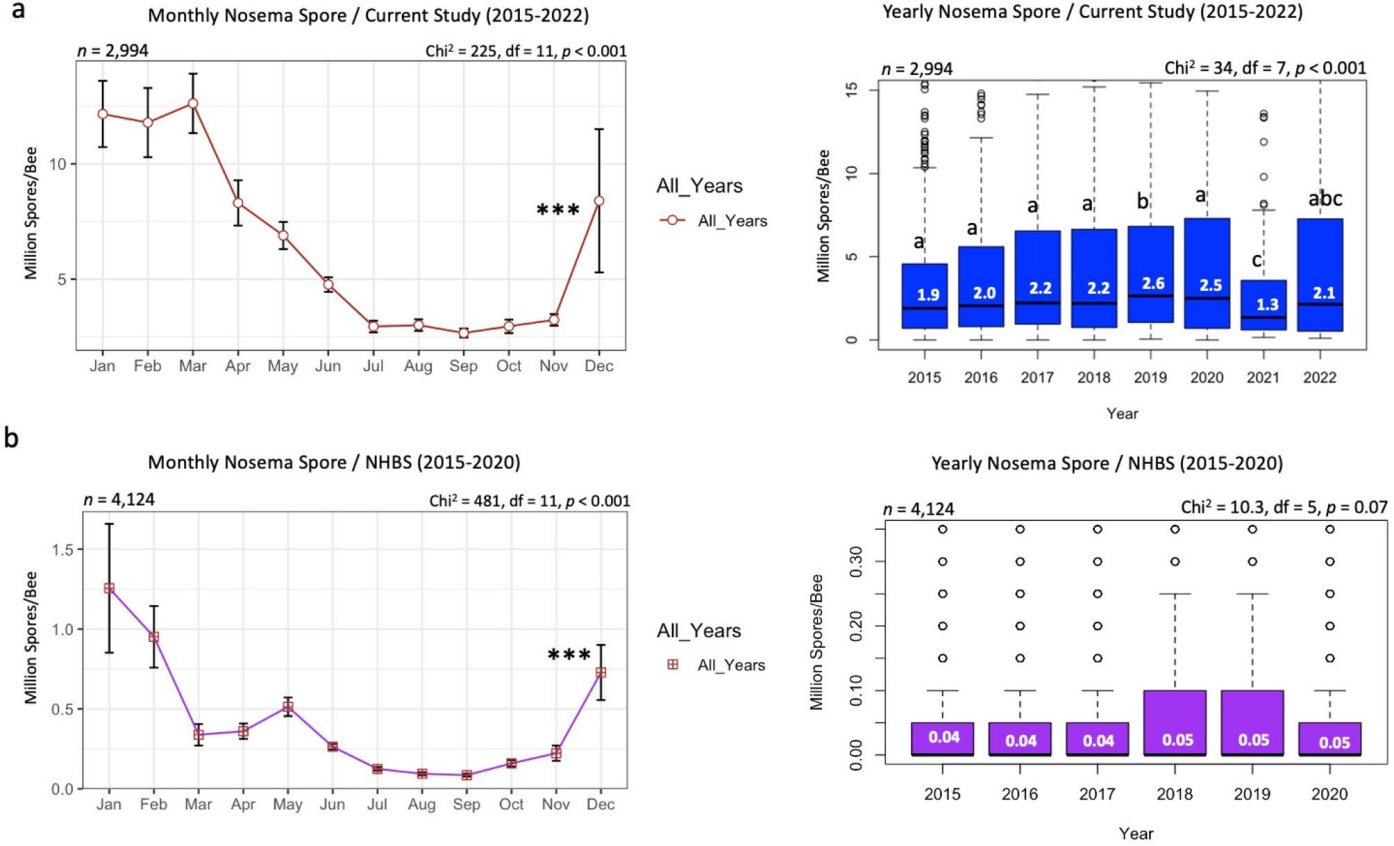
Nationwide *Nosema* spore count displayed by month and year for both datasets: Current study (**a**) and the National Honey Bee Survey NHBS (**b**). (n) is the number of samples in each graph. Error bars in the line graphs are the Standard Error (SE). The Kruskal–Wallis test was conducted at three levels of significance: *p* < 0.05*, *p* < 0.01**, *p* < 0.001***. The box plot's median is displayed in white font, and box plots with different alphabetic letters are statistically significant.

The U.S. states showed noticeable variation in *Nosema* spore count across the years (Figs. 7, 8). The national ranges for spore count per year were as follows: 2015: (0–14 Million Spores/Bee); 2016: (0–30); 2017: (0–22); 2018: (1–30); 2019: (0–102); 2020: (1–36); 2021: (0–26), and 2022: (0–25) (Figs. 7, 8). In terms of state count, in 2015, the highest spore counts were found in NH (14.33) and NJ (13.26) (Fig. 7). In 2016: NC (29.86) and MD (27.19), in 2017: WV (21.73) and CO (13.4), in 2018: MI (29.09) and MN (21.06) (Fig. 7). States with the highest spore counts in the following four years were as follows: in 2019: NH (102) and LA (20.7); in 2020: NH (36.35) and VA (15.88); in 2021: ND (25.75) and MI (15.98), and in 2022: CT (25) and MN (15.7) (Fig. 8). The national average of spore count varied significantly (*p* < 0.001) across months and ranged between 2 (July to October) to 15 million spores during the winter season. Spore counts were significantly higher in the winter season and dropped significantly from June to October (Fig. 9a). On a year-to-year basis, the lowest spore count was recorded in 2021 (1.3). Spore loads from 2015 to 2018 were steady (1.9–2.2) with no significant differences. An uptick and significant increase were recorded in 2019 (2.6). However, spore loads dropped again to previous ranges (1.3–2.5) in 2020 and 2022 (Fig. 9a). The nationwide average of *Nosema* spore count and prevalence across the U.S. were 6.8 million spores per bee and 99.73%, respectively (Table 1). The monthly trend of *Nosema* spore count from the NHBS matches the one observed in our data but with much lower rates (0.1–1.2) (Fig. 9b). NHBS data showed a steady rate of overall *Nosema* infection per year (0.04–0.05) with no significant differences (*p* = 0.07) (Fig. 9b).

### *Nosema* spore count and climate regions

**Figure 10:**
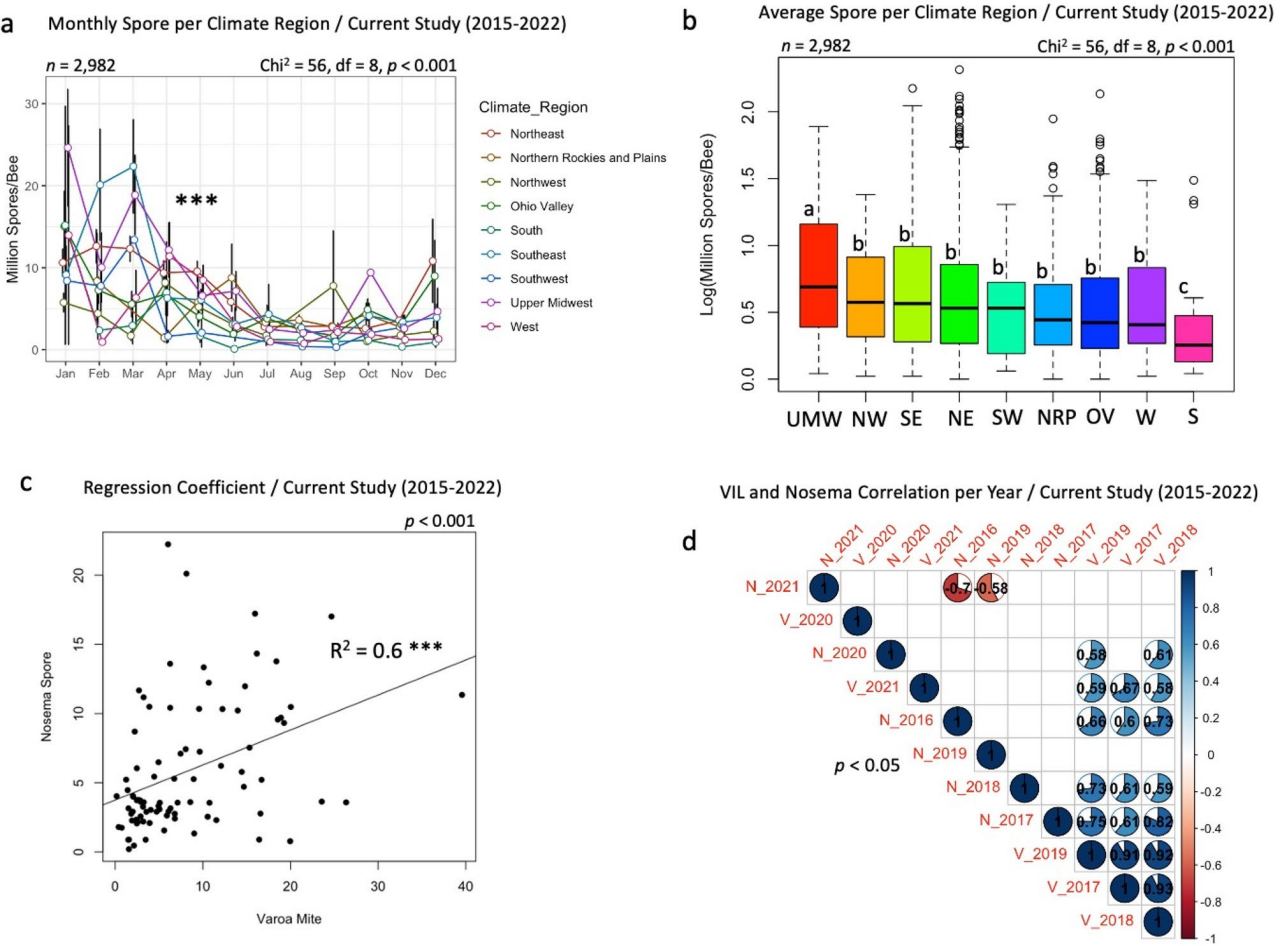
Climate region implication on *Nosema* load and correlations between VIL and *Nosema* infection. (**a**) Monthly longitudinal display of average spores per climate region. (**b**) Overall spore average per climate region. (**c**) Regression line and coefficient of *Varroa* mite load and *Nosema* spore count. (**d**) Correlation of *Varroa* mite infestation and *Nosema* infection conducted by average year load. The correlation was conducted at *p* < 0.05 cutoff, R-values are given within pairwise correlation circles, only significant correlations are displayed, and blank squares were not significant. Box plots with different alphabetic letters are statistically significant.

Analysis of *Nosema* spore load by month from different climate regions showed significant differences (*p* < 0.001) among the latter (Fig. 10a). Highest levels of infection were recorded from January to April, followed by declines from May to November and increased again in December (Fig. 10a). The highest overall *Nosema* spore count was recorded in Upper Midwest region and the lowest in the South region. No significant differences in *Nosema* load were found among the other six climate regions (Fig. 10b).

### VIL and *Nosema* relationship

A regression analysis was conducted to elaborate on the relationship between mite load and *Nosema* spore count. This analysis was carried out on national monthly averages for both variables. It showed a significant (*p* < 0.001) positive linear trend (R^2^ = 0.6) between VIL and *Nosema* infection in our analyzed samples (Fig. 10c). Similarly, multiple positive yearly correlations between VIL and *Nosema* infection were identified, as well as two negative correlations between *Nosema* loads in 2021 and both 2016 and 2019 with R = − 0.7 and − 0.6 respectively (Fig. 10d).

## Discussion

The *Varroa destructor*, considered one of the most devastating parasites and the leading cause of colony mortality, was identified in all state samples analyzed over eight years. Few previous studies from Latin American countries reported VIL-related data. In Colombia, mainly among Africanized honey bee (AHB) populations, a VIL of 4.5% was reported^34^ (Table 2). Mexico, which comprises a mix of Africanized honey bees (AHB) and European honey bees (EHB) populations^35,36^, reported VIL ranging between 3.5 and 7.4%^37,38^, Table 2. A recent longitudinal study from Brazil reviewing VIL from 1979 to 2020 within AHB populations reported a VIL of 4.5%^39^. Similarly close VIL of 5.4% was reported in the same country in another study^40^.

**Table 2:** Honey bee epidemiological data related to VIL and *Nosema* infection from different countries. (N): Number of samples or colony, (VIL): *Varroa* infestation level, (VP): *Varroa* prevalence, (SC): *Nosema* spore count in million spores per bee, (NP): *Nosema* prevalence, (GB): Predominant genetic background, (EHB): European honey bees, (AHB): Africanized honey bees, (BM): Beekeeping mode and (HS): Health status of colony.

Country	Authority	Year	N	VIL %	VP %	References	GB	BM	HS
*Varroa* mite									
USA	USDA-ARS/BRL	2015–2022	4039	8.21	85.14	Current Study	EHB	Mix	Clinically ill
USA	USDA-APHIS/NHBS	2015–2020	4860	3.09	91.52	Current Study	EHB	Mix	Healthy
USA	USDA-APHIS/NHBS	2009–2014	2901	6	97	^52^	EHB	Mix	Healthy
Argentina	SENASA	2005–2007	1896	na	74	^53^	EHB	Stationary	Healthy
Brazil	Academic Report	1979–2020	361	4.5	na	^39^	AHB	Stationary	Healthy
Brazil	Academic Report	2009–2010	438	5.4	95.7	^40^	AHB	Stationary	Healthy
Brazil	Academic Report	1986	18	7.5	na	^43^	EHB	Stationary	Healthy
Brazil	Academic Report	1986	18	5.8	na	^43^	AHB	Stationary	Healthy
Colombia	Academic Report	2013–2014	483	4.5	92	^34^	AHB	Stationary	Healthy
Mexico	Academic Report	2013	80	7.4	na	^37^	AHB/EHB	Stationary	Healthy
Mexico	Academic Report	2016	369	5.2	87.8	^38^	AHB/EHB	Stationary	Healthy
Mexico	Academic Report	2016–2017	369	3.5	83.5	^54^	AHB/EHB	Stationary	Healthy
Uruguay	Academic Report	2011	103	4.4	75.7	^55^	AHB/EHB	Stationary	Healthy

Country	Authority	Year	N	SC	NP %	Reference	GB	BM	HS
*Nosema* spp.									
USA	USDA-ARS/BRL	2015–2022	2994	6.8	99.73	Current Study	EHB	Mix	Clinically ill
USA	USDA-APHIS/NHBS	2015–2020	4124	0.21	46.56	Current Study	EHB	Mix	Healthy
Mexico	Academic	2017	369	na	48.5	^54^	AHB/EHB	Stationary	Healthy
Argentina	SENASA	2012	333	na	49	^53^	EHB	Mix	Healthy
Brazil	Academic	2009–2012	637	na	79.9	^56^	AHB	Stationary	Healthy
Chie	Academic	2012	240	0.1	49	^57^	EHB	Mix	Healthy

The U.S. national VIL found within clinically diseased colonies (8.21%) fits closer to the end range found in Mexico, which relies on both EHB and AHB populations (Table 2). Understanding the genetic background of the honey bee populations is essential and critical in this context, as AHB populations were described to be more resistant to *Varroa* mite infestation compared to EHB populations^41–43^. Arguably one of the most comprehensive U.S. data related to mite infestation and *Nosema* spore load was generated by the USDA-APHIS National Honey Bee Survey (NHBS). This survey has been conducted on healthy honey bee colonies across the U.S.A. since 2009. In order to explore further our findings, we compared our data with those of the NHBS within the same year range (2015–2020) of our current study generated at the Bee Research Laboratory (BRL). Clearly, the samples we received and analyzed reflect clinically diseased populations or colonies as both overall VIL and *Nosema* loads are significantly higher than what was found in the data from the NHBS, which was randomly conducted nationwide on healthy colonies (Figs. S1, S2).

Interestingly, *Varroa* prevalence was higher (91.52%) in the NHBS data than our BRL data (85.14%), indicating significant proliferation of this parasite regardless of the colony's health status (Fig. S1b). This was not the case for *Nosema* prevalence, which was approximately two-fold higher in our samples (99.73%) than in healthy colonies (46.54%) sampled by the NHBS (Fig. S2b). The overall VIL recorded in our diagnosed samples (8.21%) was significantly higher than the nationwide average VIL of healthy colonies reported by the NHBS (3.09%) (Fig. S1). Therefore, it is conceivable to hypothesize that elevated levels of *Varroa* mites may have triggered the decline of these colonies. Moreover, monthly *Varroa* load in diseased, weak, or struggling colonies did not follow the same patterns identified in healthy colonies (Fig. 4). For instance, samples from weak and/or dying colonies shipped to us in winter time had the highest VIL.

In contrast, the NHBS data, which displays the classic bee-*Varroa* parasitism dynamic, showed a single high peak in October to drop again during winter. Hence, the monthly VIL pattern of the diseased samples (BRL data) could reflect the intensification of *Varroa* treatments in unwell colonies during the beekeeping season (Apr–Oct), which had pushed the parasite development and survival to Winter, when beekeepers usually apply no treatment. In such struggling colonies, a take-over of the parasite has likely been established, leading to bees' failure to suppress *Varroa* mites. Such a phenomenon is supported by a constantly elevated VIL exceeding the 4% critical threshold^44^ in our samples. On the other hand, the host-parasite dynamic in healthy colonies differed from weak declining colonies as the former's social resilience and ability to suppress diseases remained intact. This is supported by the fact that colonies of the NHBS maintained a relatively low range of VIL (1–6%) across the year (Fig. 4). Eventually, such resilience towards *Varroa* mites was not due to natural resistance per se, but rather to intensive pest management control carried out by beekeepers nationwide. BRL and NHBS data firmly agreed that colonies in the West region (CA and NV) had the lowest VIL. Despite slight differences between both datasets vis-à-vis regions with the highest VIL, the consensus indicates that northern zones with colder climates favored higher VIL. Besides significant variations in VIL among climate regions, the absence of meaningful correlations of VIL among regions is strong evidence of the implication of climate region on *Varroa* mite infestation. The highest *Nosema* infestation identified in the Upper Midwest region reveals a more effective proliferation and intensification of this pathogen in cold, moist environments, as pointed out in a previous study^45^.

In conclusion, this study provides valuable information and significant insights into the seasonal variation and distribution of *Varroa* mite infestation and *Nosema* infection among diseased honey bee colonies in the U.S.A. This underscores the necessity for ongoing commitments from beekeepers and researchers to develop and implement strategies to prevent and mitigate honey bee diseases and stressors, including integrated pest management practices, breeding for disease-resistant bees, and promoting habitat conservation. Addressing honey bee disease challenges is of utmost significance to safeguard the well-being of honey bees and to uphold their pivotal role in providing essential pollination services.

## Methods

### Honey bee samples

The Bee Research Laboratory (BRL) received a total of 7,033 symptomatic honey bee samples between 2015 and 2022. Honey bee samples are usually soaked in 70% ethanol and shipped via post mail. Based on our guidelines, State Apiary Inspectors and beekeepers typically collected approximately 300 adult bees from each colony with signs of diseases, weakening or declining colonies, and in some cases, dead bees from collapsed colonies. The identification information, including the sampling date, locations (state, zip code), and names of beekeepers or apiary inspectors, were all marked on the plastic zipper bags used for holding bee samples.

### *Varroa* infestation level

The *Varroa* Infestation Level (VIL) was described for each sample based on the number of mites identified. VIL was calculated as a percentage by dividing the number of mites by the number of bees in a sample and multiplying by 100 as described in previous studies^34,46^. *Varroa* mite load per bee sample was obtained using a multistep process. Firstly, the bees in each sample were agitated and transferred into a screening container fitted with a #8 mesh insert. This mesh effectively retained the bees while allowing debris and varroa mites to pass through. The screening container was placed within a fine mesh sieve and placed underneath a tap. The tap was opened fully to pressure wash the bees for 40 s while continuously swirling the screening container. Once the washing process was completed, the screening container was set aside for water to drain off, and any remaining water was absorbed using tissue paper. The total number of bees per sample was determined based on the weight of a single wet bee (0.16129 g), which was obtained from previous trials and regression correlations conducted in our lab. Subsequently, each sample's total number of bees was calculated based on their collective weight. The mites collected on the fine mesh during the washing process were carefully counted, and VIL was calculated as described above.

### *Nosema* infection

For each colony, 30 honey bee workers were randomly subsampled and examined. Honey bee abdomens were removed and placed in a zip-lock bag. The abdomens were crushed within the bag using a rolling pin on a flat surface. Subsequently, 30 mL of distilled water was added to the bag to create a homogenous suspension. Microscopic examination was conducted on 10 µL of the suspension, which was transferred to a hemocytometer, examined under a light microscope at 400× magnification^47^, and screened for the presence or absence of *Nosema* spp. If *Nosema* spores were observed, they were quantified, and the infestation rates of *Nosema* spp. were calculated based on the number of spores counted per examined field^48^, with the results expressed as millions of spores per bee.

### VIL and *Nosema* spore loads across the U.S. climate regions

This prevalence of *Varroa* and *Nosema* is expressed as the percentage of samples that tested positive for either of these parasites within a specific state or geographic region. *Varroa* and *Nosema* prevalence were analyzed and studied from a geographical perspective based on climate regions. The classification of climate regions used in this study was based on the official U.S. government classification, as outlined by the National Centers for Environmental Information. According to this classification, the U.S. states were grouped into nine major climate regions^49^, namely: (1) Northeast (NE), (2) Northern Rockies and Plains (NRP), (3) Northwest (NW), (4) Ohio Valley (OV), (5) Southeast (SE), (6) Southwest (SW), (7) Upper Midwest (UMW) and (8) West (W). Further information regarding the specific states within each climate region is provided in Table 1, along with the corresponding abbreviations used throughout the text and figures.

### National honey bee survey NHBS data

Since 2009, the USDA-APHIS, through the National Honey Bee Survey program (NHBS), has conducted an ongoing random and regular honey bee disease screening on healthy colonies across the U.S. As a means of comparison with our data, the full range of data related to *Varroa* mite infestation and *Nosema* spore count were downloaded from the public-facing NHBS website https://research.beeinformed.org/state_reports/. This study used and analyzed datasets of the NHBS (2015–2020) matching the year range of the BDDL dataset (2015–2022). NHBS data for 2021 and 2022 were not publicly available.

### Data analysis

Statistical analyses and statistically-related figures for each dataset were carried out in the R environment^50^ via RStudio^51^ Version (2022.07.0). Geographical mapping of honey bee pests was conducted using the Tableau Public platform (https://www.tableau.com). All datasets were tested for normality using the Shapiro test before conducting statistical analyses. Kruskal–Wallis rank test, a non-parametric test, was conducted at a 95% confidential interval with three levels of significance (*p* < 0.05*;  < 0.001**;  < 0.001***) on data that failed the normality test. Adjustments to *p*-values were made where appropriate using the Benjamini–Hochberg method.

Figures were generated in the R environment utilizing three main Libraries: “ggplot2”, “doby”, and “plyr”. Mite and *Nosema* loads were displayed longitudinally per month and as overall averages per year or climate region. Error bars of all line graphs represent the Standard Error (SE) except for the boxplots (box and whisker plots), which display the median, first and third quartiles, and both maximum and minimum values of variables. Outliers from Box plot graphs were omitted in some instances with log transformation for better data visualization. Average VIL and *Nosema* spore count per state and nationwide, summarized mainly in Tables, represent the mean values and not the medians unless stated otherwise. Heatmaps were generated for VIL data using the “pheatmap” library to visualize the relationships between mite load vis-à-vis month and climate region. Correlation analyses between variables were all conducted at a cutoff of *p* < 0.05 with the “spearman” method as the data failed normality utilizing both the “corrplot” and “Hmisc” Libraries. Linear regression analysis was conducted to study the relationships between both *Varroa* and *Nosema* loads as variables.

### Supplementary Information

Supplementary Figures.

## Data Availability

The datasets generated and/or analyzed during the current study are not publicly available as they contain Personal Identifiable Information but are available from the corresponding author on reasonable request.
