# Supplementary material for: Prevalence and distribution of Varroa destructor and Nosema spp. in symptomatic honey bee colonies across the USA from 2015 to 2022
Source: Sci Rep. 2024 Jan 19;14:1726. doi: 10.1038/s41598-024-51514-9 (PMC10798951; doi:10.1038/s41598-024-51514-9)
Supplement: Supplementary file 1 — Supplementary Figures. [file 41598_2024_51514_MOESM1_ESM.docx]

**Figure S1.**  Overall *Varroa* mite infection VIL comparison between both databases: 1- Bee Research Laboratory data (Current study) and 2- The National Honey Bee Survey NHBS. (a): Overall VIL average with medians displayed, (b): Percentage of overall *Varroa* mite prevalence with means displayed.

**
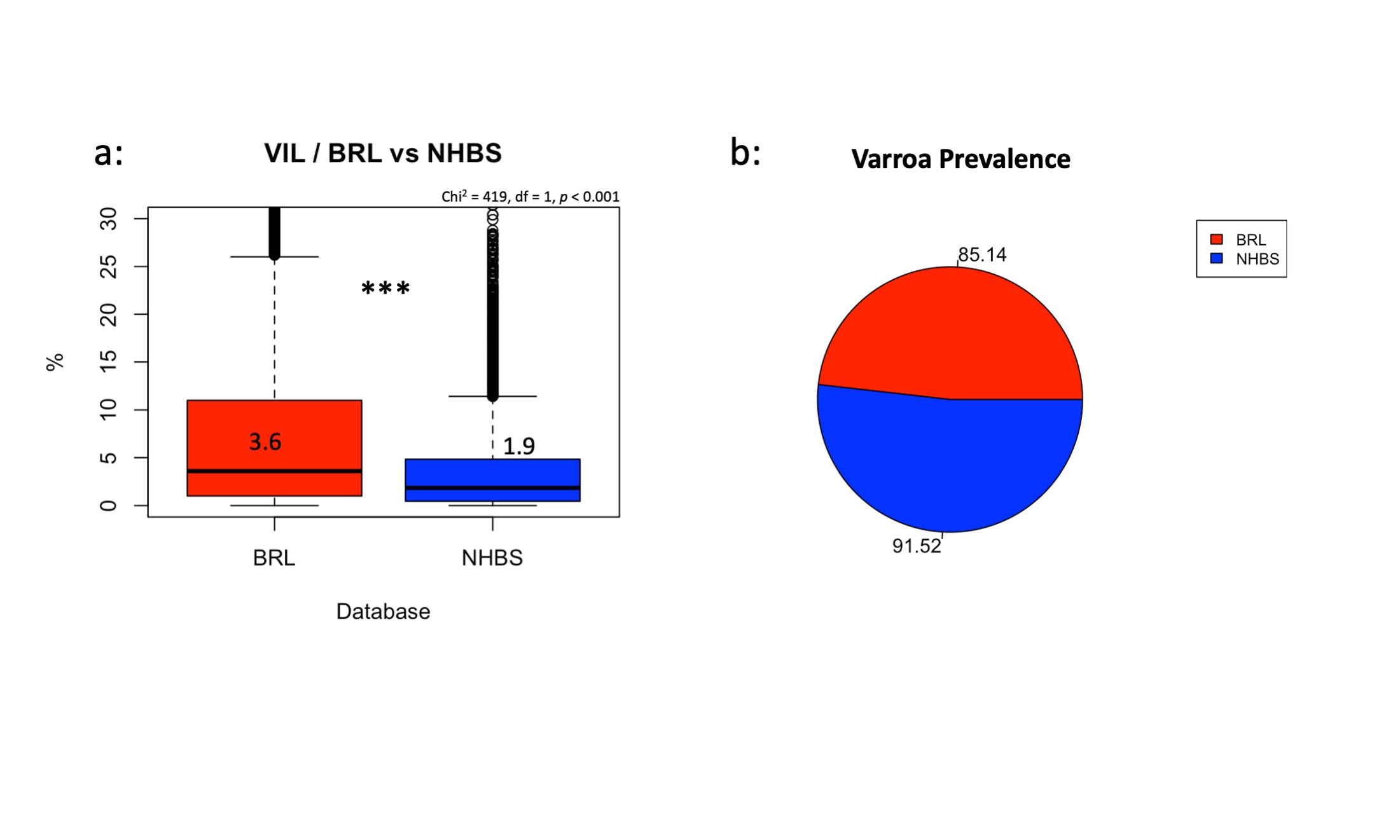
**

**Figure S2.**  Comparison of overall *Nosema* spore load between both databases: 1- Bee Research Laboratory data (Current study) and 2- The National Honey Bee Survey NHBS. (a): Overall averages of *Nosema* spore count (Million spore/bee) with medians displayed, (b): Percentage of overall *Nosema* prevalence with means displayed.

**
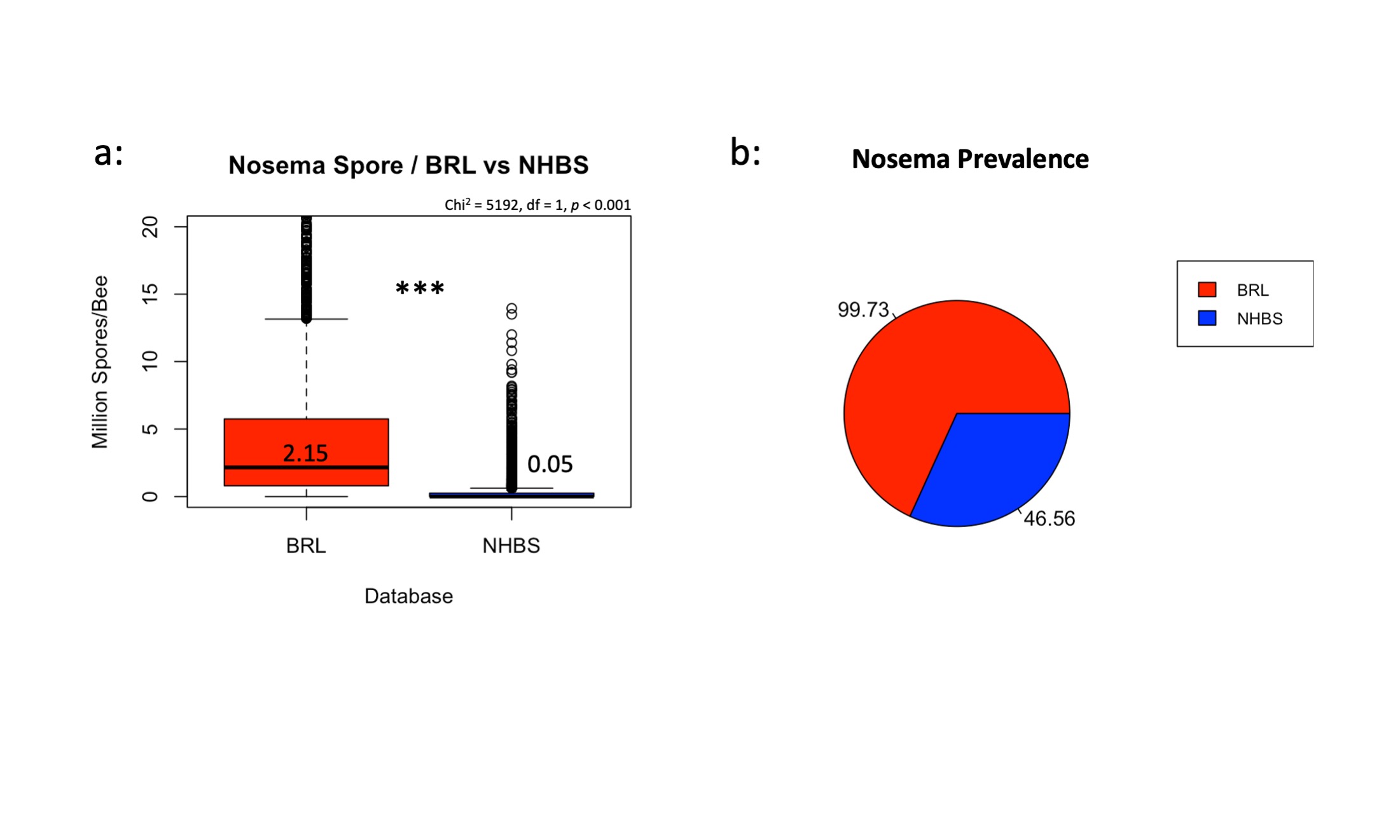
**
